# Differential Protein Expression in Sugarcane during Sugarcane-*Sporisorium scitamineum* Interaction Revealed by 2-DE and MALDI-TOF-TOF/MS

**DOI:** 10.1155/2011/989016

**Published:** 2011-08-01

**Authors:** Youxiong Que, Liping Xu, Jianwei Lin, Miaohong Ruan, Muqing Zhang, Rukai Chen

**Affiliations:** Key Lab of Sugarcane Genetic Improvement, Ministry of Agriculture, Fujian Agriculture and Forestry University, Fuzhou, Fujian 350002, China

## Abstract

To understand the molecular basis of a specific plant-pathogen interaction, it is important to identify plant proteins that respond to the pathogen attack. Two sugarcane varieties, NCo376 and Ya71-374, were used in this study. By applying 2-dimensional electrophoresis (2-DE), the protein expression profile of sugarcane after inoculating with *Sporisorium scitamineum* was analyzed. In total, 23 differentially expressed proteins were identified by MALDI-TOF-TOF/MS. Bioinformatics analysis revealed that the functions of these 20 differential proteins were associated with such functions as photosynthesis, signal transduction, and disease resistance, while the function of the remaining three proteins was not determined. From above, we can assume that the protein regulatory network during the interaction between sugarcane and *S. scitamineum* is complicated. This represents the first proteomic investigation focused on highlighting the alterations of the protein expression profile in sugarcane exposed to *S. scitamineum*, and it provides reference information on sugarcane response to *S. scitamineum* stress at the protein level.

## 1. Introduction

Sugarcane smut, which causes significant losses in cane and sugar yield as well as lowering sucrose content, is a fungal disease caused by *Sporisorium scitamineum*. Following infection, sugarcane plants often tiller profusely with the shoots being more spindly and the leaves being more upright and narrow (“grass-like” in appearance) and “buggy-like” whips emerging from the shoots. Less common symptoms are leaf and stem galls and proliferating buds. The development of sugarcane smut depends on environment, cultivar, and pathogen interactions.

Plant disease resistance is complex, involving a complex network of recognition of avirulence determinants by plant receptors, triggering of specific signal transduction pathways, oxidative bursts, accumulation of pathogenesis-related (PR-) proteins and phytoalexins, and localized cell death [1]. Understanding the basis of pathogens causing a certain disease in one host plant but not in another has long intrigued and motivated plant pathologists. Until recently, increasing attention has been paid to the study of the interaction between sugarcane and *S. scitamineum*. The research reports have mainly concentrated on the physiological and biochemical changes during interaction between sugarcane and *S. scitamineum*, which should be helpful in accelerating the formulation of short- and long-term strategies of smut disease management [2–4]. Additionally, the study of sugarcane-*S. scitamineum* interaction at the molecular level has also identified an inventory of candidate genes that are preferentially expressed during the process [5–7], suggesting an active role for the host plant. 

Despite what we have already learned, little is known about the proteomic background of the interaction between pathogen and host in this pathosystem. Differential proteomics, whose essence is to discover the differential protein expression among different samples caused by a specific factor, is an important part of the overall proteomics study. The identification of differentially expressed proteins under various exogenous stresses can most probably give clues as to what kind of defensive mechanisms and biochemical pathways are regulated in specific circumstances. Theoretically, once enough information about protein differences is obtained, the basis for these changes can be deduced; thus, the study of differential proteomics provides a powerful tool for basic life science research. Recently, methods to study proteins that show differences in abundance under different conditions, such as 2-DE and MALDI-TOF-TOF/MS, have been developed. Moreover, the protein expression profiles of many plants under various biotic stresses have been studied by the application of differential proteomics technology, and the relevant information of the response mechanism of differential protein under various stresses has been obtained [8–12]. Thus, this technology has been demonstrated to be an excellent tool to identify novel proteins related with plant resistance to certain pathogen. 

In the present study, sugarcane varieties NCo376 and Ya71-374, which were highly resistant and highly susceptible to *S. scitamineum*, respectively, were used as plant materials, and two kinds of differential proteomics technology, 2-DE and MALDI-TOF-TOF/MS (Matrix Assisted Laser Desorption Ionization, tandem Time of Flight Mass Spectrometer), were applied in the differentially expressed proteins analysis during sugarcane-*S. scitamineum *interaction. It is anticipated that the results obtained in this study will be helpful toward understanding the molecular response of sugarcane varieties with different smut resistance levels to *S. scitamineum*, and it will thus provide some information on sugarcane smut resistance basis at the protein level.

## 2. Materials and Methods

### 2.1. Plant Materials and Inoculation

Sugarcane varieties, NCo376 (highly resistant to *S. scitamineum*) and Ya71-374 (highly susceptible to *S. scitamineum*), were used and the source of *S. scitamineum* Race 2 inoculum was collected from sugarcane variety F134. Thirty stalks each of NCo376 and Ya71-374 with robust and uniform growth were selected, cut into two-bud stalk, and divided into two groups of equal sections. The stalks were treated in hot water at 50°C for 2 hours for disinfection. One group of the disinfected stalks was immersed in *S. scitamineum* spore suspension for 10 min (treatment group), and the other group was treated with sterilized double-distilled water (control group). The concentration of the *S. scitamineum* spore suspension used for inoculation was 5 × 10^6^ spores/mL. After inoculation, stalks were kept under humid condition at 25°C for 24 hours and then planted in the field. For the control group, the stalks were treated the same as the treatment group except that the *S. scitamineum* spore suspension was replaced by distilled water. When symptoms of *S. scitamineum* infection appeared, the +1 leaves (the 1st leaf under the fleshy band) in the treatment and control groups were collected by freezing in liquid nitrogen and stored at −80°C until later use for protein extraction. Three biological repeats were applied in the experiment.

### 2.2. Extraction of Whole Protein from Sugarcane Leaves

TCA-acetone (trichloroacetic acid and acetone) precipitation method was adopted for the extraction of whole protein with minor modification [13]. Firstly, 1 g of fresh sugarcane leaves was grinded into powder in liquid nitrogen (PVP: sample was 1 : 10) with the addition of 10 mL of 10% TCA (prepared by acetone, containing 0.07%  *β*-mercaptoethanol). Secondly, the mixed liquid was precipitated for 1 h at −20°C and centrifuged for 15 min with 30000 g at 4°C, and then the supernatant was discarded and the pellet was re-suspended with cold acetone containing 0.07%  *β*-mercaptoethanol and kept overnight at −20°C; after that, the pellet was washed twice with cold acetone containing 0.07%  *β*-mercaptoethanol. Thirdly, after centrifugation for 15 min with 30000 g, the pellet was resuspended with 80%  acetone and kept for 1 h at −20°C, and then it was centrifuged again. Finally, the pellet was dried into powder by vacuum drying at low temperature and stored at −80°C until use.

### 2.3. Protein Lysis and Content Determination

50 mg of protein dry powder was added into 600 *μ*L lysate (7 mol/L urea, 2 mol/L TCA, 4% (m/v) 3-((3-cholamidopropyl) dimethylammonio-1-propanesulfonate (CHAPS), 1% (m/v) ampholyte with pH of 3.5–10, and 40 mmol/L dithiothreitol (DTT)), and lysed in water bath for 2 h at 30°C, and then 20,000 gcentrifuged was for 20 min. The supernatant was used for 2-DE analysis. Total protein content was measured according to the method developed by Bradford (1976) using bovine serum albumin as the standard [14]. 

### 2.4. First-Dimension Isoelectric Focusing (IEF)

Immobilized pH gradient (IPG) strip (24 cm, pH 4–7, linear gradient; Amersham Biosciences Company) was applied with first-dimensional isoelectric focusing (IEF) on Amersham Ettan IPGphor II. Before use, the protein extracts were allowed to thaw at room temperature. Active rehydration was performed, and the rehydration solution contained 6 mol/L of urea, 2 mol/L of thiourea, 2% CHAPS, 0.5% IPG buffer, 0.4% DTT, and 0.002% bromphenol blue. The total sample volume was 450 *μ*L, containing 1,000 *μ*g protein. When the sample was added into the gel-holding tank, the plastic protective film on the strip was taken off and the strip surface faced downward and both ends of the strip clung to the electrodes at the bottom of the tank. Attention should be paid that the positive and negative electrodes not be reversed and the generation of bubble between strip and sample fluid be avoided. 1.5 mL of mineral oil was covered on the strip to prevent volatilization of the sample solution. The working parameters for IEF were as follows: 20°C, 50 *μ*A/strip, 30 V, 12 h; 500 V, 1 h; 1,000 V, 2 h; 3,000 V, 1 h; 8,000 V, 7.5 h, of which a total of 65, 860 V hours were achieved.

### 2.5. Second-Dimension SDS-PAGE

When IEF was complete, the strips were incubated for 15 min in the equilibration buffer (6 mol/L of urea, 50 mmol/L Tris-HCl (pH 8.8), 2% (m/v) SDS, 30% (m/v) glycerol, 0.002% bromphenol blue) containing 1% (m/v) DTT, and then the strips were reequilibrated for 20 min in the equilibration buffer which contained 2.5% iodoacetamide to remove the surplus DTT. After equilibrating twice, the second-dimensional SDS-PAGE was adopted with the gel concentration of 12.5% T and 2.6% C. After the solidification of the gel, the strips were placed into the glass plate carefully with good contact with the gel surface (avoiding the generation of bubble), and 0.5% agarose containing a trace amount of bromphenol blue (prepared by electrophoresis buffer) was used for gel sealing, electrophoresis was started, and the parameter was set as 15 mA/plate. When the front edge of bromphenol blue reached the gel, the electric current was increased to 30 mA/plate and the electrophoresis was stopped when the front edge of the bromphenol blue reached the position 0.5 cm away from the bottom of gel. Once the SDS-PAGE finished, the gel was taken out and stained with the Neuhoff colloid staining method [15]. Gel image was acquired by a Gel Doc 2000 (Bio-Rad) image scanner and spot detection, spot matching, and quantitative intensity analysis were performed using PD-Quest 7.20 software (Bio-Rad). Images of treatment and control gels were normalized through intensity normalization, and only those proteins with obvious upregulated expression or significant downregulated expression and new proteins after infection were considered to be differentially expressed.

### 2.6. In-Gel Digestion of Differentially Expressed Proteins

Proteins of interest were excised from the gels and placed into a 96-well microtitre plate. Gel pieces were detained with a solution of 15 mmol/L potassium ferricyanide and 50 mmol/L sodium thiosulfate (1 : 1), for 20 min at room temperature. After that, they were washed twice with deionized water, and they shrank by dehydration in acetonitrile (ACN). The samples were then swollen in a digestion buffer containing 20 mmol/L ammonium bicarbonate and 12.5 ng/*μ*L trypsin at 4°C. After 30 min incubation, the gels were digested more than 12 h at 37°C. Peptides were then extracted twice using 0.1% TFA in 50% ACN. The extracts were dried under the protection of N_2_. For MALDI-TOF-TOF/MS, the peptides were eluted onto the target plate with 0.7 *μ*L matrix solution (*α*-cyano-4-hydroxy-cinnamic acid in 0.1% TFA and 50% ACN). 

### 2.7. MALDI-TOF-TOF/MS and Data Analysis

Samples were analyzed by a 4700 Proteomics Analyzer (MALDI-TOF/TOF TM) (Applied Biosystems, USA), and the operations were as follows. The completely dry peptide segments were dissolved in 0.7 *μ*L of 0.5 g/L CHCA solution (0.1% TFA + 50% ACN solvent). The entire solution was spot onto the stainless steel MALDI target plate with air drying at room temperature. Then mass spectrometric analysis was performed on the samples, and the laser light was from Nd : YAG laser with a wavelength of 355 nm with an accelerating voltage of 20 kV. The data was collected by positive ion and automated acquisition mode. The scope of PMF mass scan was from 700 D to 3500 D, and series mass spectrometric analysis was made on 5 peaks with the maximum intensity. External standard adjustment was made by myoglobin enzymolysis peptide segment on the spectrogram. 

The data were searched by GPS Explorer (GPS Explorer TM software, Applied Biosystems, USA) using MASCOT (Matrix Science, London, UK) as a search engine. Setting of parameters was as follows: database was NCBI (nr); retrieval genera was set as all; data acquisition method was set as combined; maximum missing cut site allowed was set as 1; enzyme was set as trypsin. Setting of quality error scope was PMF 0.3 D; MS/MS 0.4 D; pancreatin self-degradation peak and pollutant peak were rejected manually during the database retrieval. 

## 3. Results and Analysis

### 3.1. DE Analysis of Differentially Expressed Proteins in Sugarcane under *S. scitamineum* Stress

**Figure 1:**
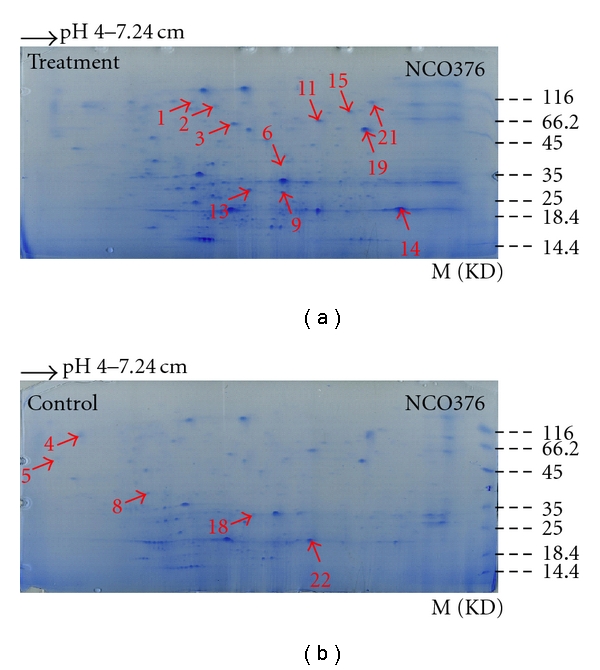
2-D SDS-PAGE proteins in NCo376 from treatment (a) and control (b) sugarcane.

**Figure 2:**
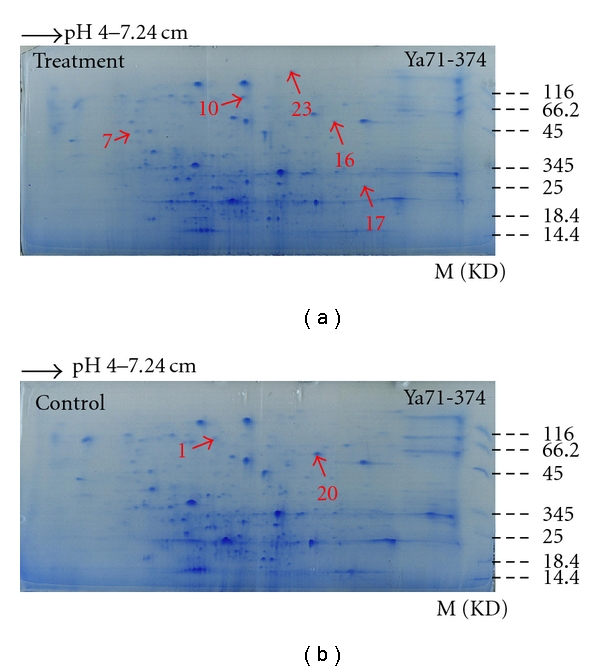
2-D SDS-PAGE proteins in Ya71-374 from treatment (a) and control (b) sugarcane.

By the application of IPG strip (pH 3–10), the result showed that the whole protein from sugarcane mainly concentrated between sub-acidity isoelectric points and neutrality, while there were only few extremely acidic proteins with isoelectric points below 4 and basic proteins with isoelectric points above 8. From the comparison of protein 2-DE atlas of highly resistant variety NCo376 and highly susceptible variety Ya71-374 before and after inoculation, we could see that the protein expression between resistant and susceptible sugarcane varieties presented some difference, which was the protein expression of the same variety before and after inoculation and the protein expression between resistant and susceptible varieties were different. While the PI (isoelectric point) of sugarcane proteins mainly concentrated between subacidity and neutrality, IPG strip with pH 3–10 could not well separate the sugarcane proteins; thus IPG strip with a narrow pH range with pH 4–7 was further applied for 2-DE analysis. Through 2-DE and silver staining, protein 2-DE gel atlas was obtained and conducted with spectrum analysis by protein discrimination software. In order to assure reproducibility, 2-DE for the control and *S. scitamineum*-stressed sugarcane was repeated three times. Image analysis showed that these 2-DE images were highly reproducible, and 2-DE patterns for control and treatment groups are shown in Figures 1 and 2. A total of about 500 to 700 visible protein spots were observed on each 2-DE gel. 

 From Figures 1 and 2, the separation effect of IPG strip (pH 4–7) on sugarcane protein was comparatively better, and we could see that no matter if in high-resistance variety NCo376 or in high-susceptibility variety Ya71-374, the protein expression presented significant difference before and after inoculation, and the difference appeared not only in the expression capacity of the same protein but also in some newly induced proteins (present) or totally inhibited (absent) proteins. At the same time, the quantity and spatial distribution of differential proteins in resistant and susceptible varieties were different, which indicated different molecular responses to *S. scitamineum* challenge. It indicated that the expression of proteins was regulated by different function modes, which resulted in the resistance of resistant variety or the susceptibility of susceptible variety to smut. 

**Figure 3:**
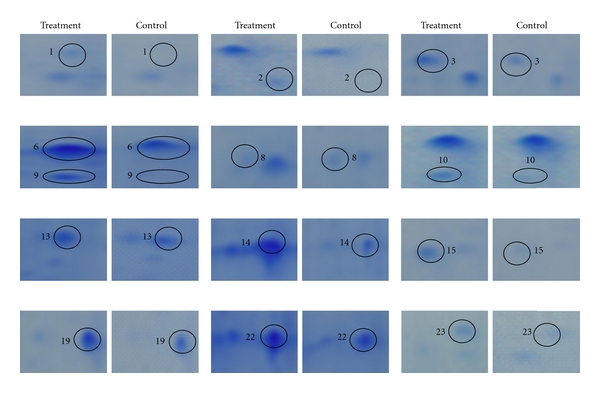
Enlarged images of partial differentially expressed proteins derived from 2-DE gel image for treatment and control sugarcane. Notes: Boxed regions show the protein, which is differentially expressed, and the number is consistent in Figures 1 and 2.

In the present study, it was observed that the expression of 23 proteins, 16 in NCo376 and seven in Ya71-374, had changed obviously before and after the *S. scitamineum* inoculation. Among these proteins, the expression amount of eight proteins (no. 2, no. 3, no. 6, no. 9, no. 11, no. 13, no. 14, and no. 19) increased and that of six proteins (no. 4, no. 5, no. 8, no. 18, no. 21, and no. 22) decreased plus two proteins newly induced after infection (no. 1, and no. 15) in NCo376, while in Ya71-374, the expression of three proteins (no. 7, no. 16, and no. 17) increased, the expression of three proteins (no. 12, no. 20, and no. 23) decreased, and one protein was newly induced after infection (no. 10) as shown in Figures 1, 2, 3, and Table 1. These differentially expressed proteins were separately excised by a clean penknife for subsequent MALDI-TOF-TOF/MS analysis. 

### 3.2. MALDI-TOF-TOF/MS Analysis of Differentially Expressed Proteins

**Figure 4:**
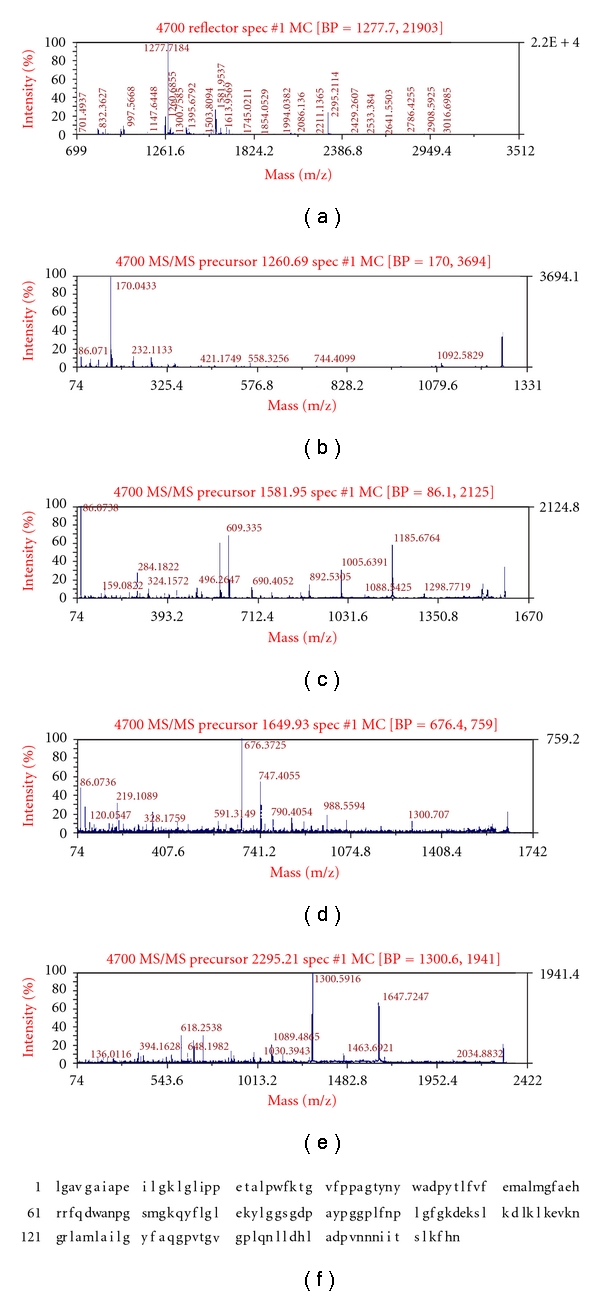
Peptide mass fingerprinting (a), tandem mass spectra (b, c, d, e), and homologous amino acid sequence (f) of chlorophyll-a/b-binding protein.

**Figure 5:**
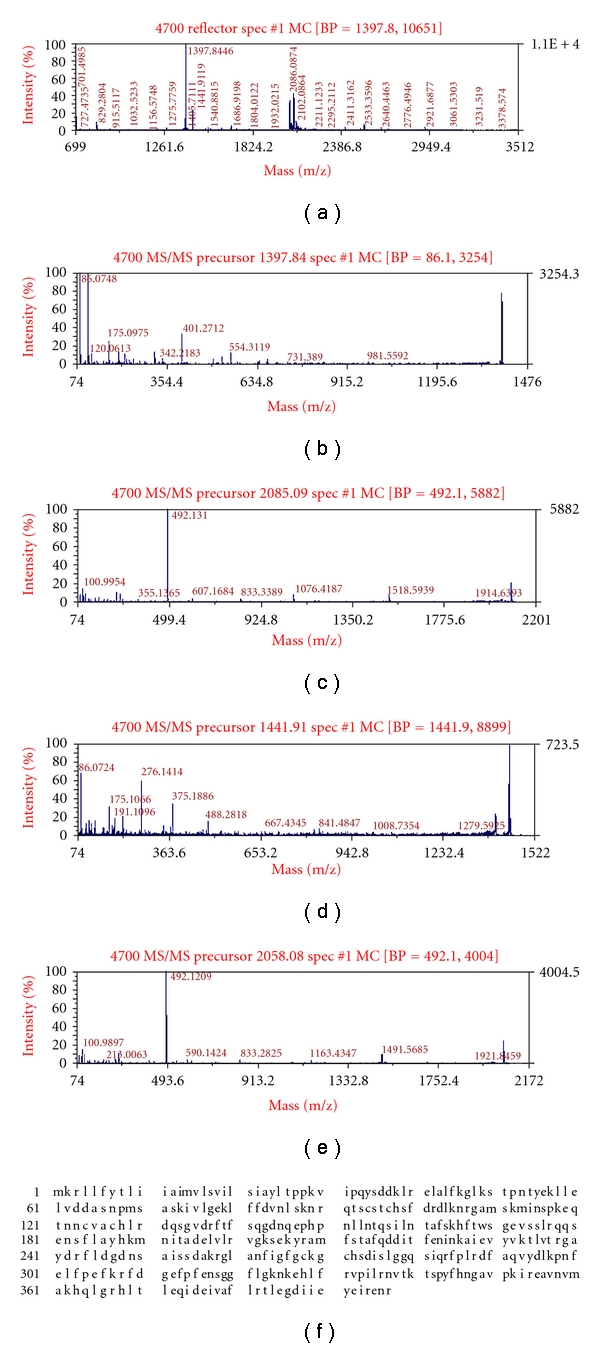
Peptide mass fingerprinting (a), tandem mass spectra (b, c, d, e), and homologous amino acid sequence (f) of cytochrome-c peroxidase protein.

In the MALDI-TOF-TOF/MS analysis of 23 differential proteins, the peptide mass fingerprinting and tandem mass spectra of 20 proteins were successfully obtained. The utility peak was not detected in three proteins numbered as 21, 22, and 23, which may be caused by their low abundance or some unknown factors during sample preparation, or because the peak value was too low to obtain the peptide mass fingerprinting. For the other 20 differential proteins, they were successfully identified in MALDI-TOF-TOF/MS analysis. Bioinformatics analysis demonstrated that the functions of these proteins were associated with such functions as photosynthesis, signal transduction, and disease resistance. Among them, a total of nine photosynthesis-related proteins constituted the largest proportion and the proportion was 45%; five were disease-resistance-related proteins that accounted for 25%; one signal-transduction-related protein accounted for 5%, while there were also three function-unknown proteins accounting for 15% of the total 20 identified proteins. Figures 4 and 5 showed the peptide mass finger printings, tandem mass spectra, and homologous amino acid sequences of two proteins (no. 9 and no. 10). 

## 4. Discussion

During the process of plant development, sugarcane is frequently infested by pathogens (including bacteria, fungi, and viruses) which are the major biotic stresses. Regardless of whether the interaction between plant and pathogen is a disease-resistant (non-affinity interaction) or susceptible reaction (affinity interaction), it is due to the interaction between the disease-resistance gene of host plant and corresponding avirulence gene of pathogen, which could induce the result of the coordination expression of a series of defense genes in host plant. However, in the reactions of affinity interaction or non-affinity interaction, the differential genes had significant differences in spatial distribution, expression rate, and expression intensity [16]. Therefore, these differences at the level of gene expression directly appeared in the various generation rate, intensity and spatial distribution of proteins in disease-resistant and susceptible varieties after the interaction between plant and pathogen [8, 17]. 

Until now, the studies on plant proteomics mainly concentrated on several plant species with completed genomic sequencing, especially on the model plants such as *O. sativa* and *A. thaliana*. Up to now, identification of protein functions continues to depend on functional genomics studies at the gene level, especially the study of ETS function. With the implementation and successful completion of sugarcane EST project, about 290,000 expressed sequence tag (EST) sequences in sugarcane have been released, but these ETSs could not yet fully cover the large genome of sugarcane, and the functions of most ESTs obtained have not yet been elucidated. Therefore, these data are far away from the requirement of protein mass spectra identification, while application of protein database of related species under the same or similar stress (such as fungi) can improve the corresponding success rate [18]. However, there are still three proteins in this study whose function has not been annotated. In recent years, with the increasing protein expression profile study of different plants under various physiological and ecological conditions, though many new proteins that related with plant-microorganism interaction were dug out, there was little research aimed at studying the function and the model of these proteins. We found that only Lee et al. (2004) used proteomics technology and identified one calcium-dependent membrane protein in salt response of root microsomes of *A. thaliana*, and through the application of reverse-genetic approach, they confirmed that this protein could mediate osmosis stress and ABA signal transduction by calcium-dependent mode [19].

To our knowledge, this is the first report focused on highlighting the alterations of the protein expression profile in sugarcane exposed to *S. scitamineum*. Investigation on proteomic aspect of interaction between sugarcane and *S. scitamineum* has the ultimate aim of providing information that may be useful for the development of the effective sugarcane smut disease management system. In the present study, the proteomic profiling techniques, 2-DE coupled with MALDI-TOF-TOF/MS, were used to analyze the differentially expressed proteins in sugarcane during sugarcane-*S. scitamineum* interaction. This approach enables direct qualitative and quantitative analysis of differentially expressed proteins during the period of disease development. In total, 23 differentially expressed proteins were obtained, among which 20 were successfully identified in MALDI-TOF-TOF/MS analysis. According to mass spectra identification and bioinformatics analysis, these proteins participated in several kinds of metabolism pathways, such as protein synthesis, signal transduction, and photosynthesis. In addition, there were also five proteins that played other functions in the interaction between sugarcane and *S. scitamineum*, and the function of the remaining three was not determined. These proteins can be divided into the following groups.

### 4.1. Protein-Synthesis-Related Proteins

HSP is a group of specific proteins of organism (or isolated culture cells) which can be induced by high salt, ABA stress, and heavy metal contamination and play a role of molecular chaperones [20]. Osmotins, whose expression is related to drought resistance, salt tolerance, and plant disease resistance, is a kind of newly synthesized or increased protein when plant faces osmosis stress [21]. Besides, the accumulation of osmotins may be a kind of elementary immune reaction generated by plants for original immune response, and osmotins may even act as the dehydration storage protein which also possesses the antifungal activity [22]. From above, the expression increase of two proteins, HSP (No.15) and osmotin (No.13), may protect cellular structure and play a role in the repair of cellular dysfunction during sugarcane-*S. scitamineum *interaction.

### 4.2. Signal Transduction Proteins

Nucleotide-binding-site (NBS) type resistance proteins are the encoding products of NBS disease-resistance genes in plants. They have kinase activity and play a similar role of transcription signal factor and activate the expression of downstream disease-resistance genes [23]. They can also activate kinase or G protein, participate in protein phosphorylation, and magnify disease-resistance response signal, and then the plant generates hypersensitive response to pathogen, which plays an important role in disease-resistance response [24, 25]. In this study, the generous expression of NBS protein termed No.19 in high-smut-resistance sugarcane may just be the basis for its high-disease-resistance. On one hand, it may play a role of smut-resistance directly; on the other hand, it may also function as kinase, and activate the expression of downstream disease-resistance genes and thus the sugarcane smut-resistance.

### 4.3. Photosynthesis-Related Proteins

Photosynthesis is one of the most important physiologic processes of plant, which plays a decisive role in the growth rate of plant, especially for C4 crop. Rubisco is a key enzyme in photosynthesis. It can regulate photosynthesis and light respiration and decide net photosynthesis [26]. Previous studies showed that RubisCO not only had organ specificity but could also be induced by many exogenous factors such as salicylic acid treatment and salt and drought stress [27]. *CAB* gene is an important gene in plant photosynthetic system, whose encoding chlorophyll-a/b-binding protein can bind with pigment and form pigment protein complex that can catch light energy, transmit the energy to reaction center quickly, drive photochemical reaction, and thus play an important role in light protection and adaption to various environments [28]. Moreover, Rieske Fe-S precursor protein is an indispensable constituent of chlorophyll body photosynthesis transfer chain. The up-regulated expression of this protein may relate to accumulated H_2_O_2_ in chlorophyll body and change the redox state of chlorophyll body, which may be a kind of response of Ya71-374 to *S. scitamineum* stress at the protein level. When smut-susceptible sugarcane variety, Ya71-374, was infected with *S. scitamineum*, the leaves turned from green to black and the main stems and branch stems grew out in the form of dust-brand lash, which resulted in plant death. This indicated that the infection of *S. scitamineum* affected the normal function of plant chlorophyll. However, when high-smut-resistance sugarcane variety, NCo376, was infected with *S. scitamineum*, the leaf surface had no disease symptom, which indicated that its photosynthesis was not affected significantly. In this study, the expression of nine photosynthesis-related proteins (No.2, No.3, No.6, No.7, No.9, No.11, No.14, No.16, and No.17) was upregulated, which most probably suggested that during the interaction between sugarcane and *S. scitamineum*, in order to defend the pathogen challenge, the expression of photosynthesis-related proteins, which was favorable for the maintenance and repair of the photosynthetic system, was upregulated. Further, the growth potential and thus the growth of sugarcane plants were improved, and the health status of the plants was maintained, which in turn increased smut resistance.

### 4.4. Other Functional Proteins

They include five proteins (No.1, NO.5, NO.8, NO.10, and NO.12). MAPs stands for tubulin, nonspecific binding nucleic acid and the proteins which play an assistant effect on microtubule function during protein-protein interaction. He et al. (2006) found that MAPs played an important role in the formation of fiber primary wall [29]. It was supposed that the newly induced expression of MAPs in sugarcane (No.1) was helpful for resisting the challenge of *S. scitamineum*. Cytochrome c peroxidase is a key enzyme during the synthesis of phytoalexin which has some inhibitory effect on disease [30]. In this study, cytochrome c peroxidase termed No.10 was newly induced after infection, and the author believed that hydrogen-peroxide-redox-type cytochrome c reaction (2 cytochrome c (Fe^2+^) + H_2_O_2_ + 2H^+^→ 2 cytochrome c (Fe^3+^) + 2H_2_O) was catalyzed by the upregulated expression of cytochrome c peroxidase, which improved the increasing synthesis of phytoalexin and inhibited the growth of *S. scitamineum* and thus reduced the harm of *S. scitamineum*. The functions of the other three proteins (NO. 5, No. 8, and No. 12) still need to be confirmed. 

### 4.5. Function-Unknown Proteins

They include a total of three proteins which termed as No. 4, No. 18, and No. 20.

 From all the above, it is presumed that there is a complicated protein regulatory network during the interaction of sugarcane-*S. scitamineum*. The regulatory network of these proteins could be just as follows: when challenged by *S. scitamineum*, sugarcane NBS-type proteins receive and transmit the stress signal, activate the expression of defense response proteins, and thus promote the synthesis of HSP and osmotins; the differential expression of photosynthesis related proteins accelerates the growth velocity of sugarcane, along with the expression of other functional resistance proteins. In all, a series of differentially expressed proteins form a closely associated regulatory network by the coupling of various endogenous signaling molecules and correlated metabolic pathways, which increase the resistance of sugarcane to *S. scitamineum*.

## 5. Conclusions

The present study reports the differential protein expression in sugarcane in response to *S. scitamineum* infection revealed by 2-DE and MALDI-TOF-TOF/MS. The results showed that there were significant differences in protein 2-DE atlas between resistant and susceptible variety, and also between the inoculated and the control sugarcane. In total, 23 proteins, including 11 upregulated, nine downregulated, and three newly induced after infection, were identified by MALDI-TOF-TOF/MS. The corresponding protein peptide mass finger printing and tandem mass spectra of 20 out of these proteins were successfully obtained. Bioinformatics analysis revealed that the functions of these 20 differential proteins were related with photosynthesis, signal transduction, disease resistance, and so on, while the function of the remaining three proteins was not determined. From above, it is assumed to be a complicated protein regulatory network during the interaction between sugarcane and *S. scitamineum*. This is the first proteomic investigation report focused on highlighting the alterations of the protein expression profile in sugarcane exposed to *S. scitamineum*. This study enriches the protein basis for sugarcane response to the infection of *S. scitamineum *and thus provides reference information for sugarcane response to *S. scitamineum* stress at the protein level, but the interrelation within each functional protein group and among functional protein groups still needs further study. Furthermore, time-consuming efforts need to be made so that more differential proteins can be identified and individual protein be investigated over the duration of the interaction, from initiation to termination.

## Figures and Tables

**Table 1:** Identification of differentially expressed proteins in sugarcane under *S. scitamineum *stress.

Spot	Accession no.	Homologous protein	pI	Mr (Da)	Species	Expression
NO. 1	gi|73970162	Echinoderm-microtubule-associated protein	8.46	233439.8	*Z. mays*	+
NO. 2	gi|3914465	Photosystem I reaction center subunit VI	10.1	14920.1	*Z. mays*	↑
NO. 3	gi|11134057	Oxygen-evolving enhancer protein 3-1	9.77	23118.5	*P. sativum*	↑
NO. 4	gi|46109064	Unknown	4.7	84190.8	*O. sativa*	↓
NO. 5	gi|8996050	Protective antigen	6.02	62682	*A. thaliana*	↓
NO. 6	gi|66821923	Ribulose-1,5-bisphosphate carboxylase	5.95	48907.6	*O. sativa*	↑
NO. 7	gi|6578770	Rubisco large subunit	6.3	48203.2	*L. cuneifolia*	↑
NO. 8	gi|89347149	2-oxo-acid dehydrogenase E1 component	5.79	95280.7	*S. bicolor*	↓
NO. 9	gi|37780998	Chlorophyll-a/b-binding protein	7.03	18027.3	*V. vinifera*	↑
NO. 10	gi|78777782	Cytochrome-c peroxidase	8.39	45113	*Z. mays*	+
NO. 11	gi|27883935	Ribulose-1,5-bisphosphate carboxylase	5.98	20501.5	*C. erectus*	↑
NO. 12	gi|75817913	Predicted oxidoreductases	6.73	11799	*A. thaliana*	↓
NO. 13	gi|62734371	Abscisic acid and stress-induced protein	6.2	15455.5	* O. sativa *	↑
NO. 14	gi|548603	Photosystem I reaction center subunit II	9.81	21919.4	*H. vulgare*	↑
NO. 15	gi|51459711	DnaK-like chaperone protein HscA	5.56	65281.9	* Z. mays*	+
NO. 16	gi|6691487	Oxygen-evolving complex of photosystem II	8.61	28121.2	*C. sativus*	↑
NO. 17	gi|50508582	Rieske Fe-S precursor protein	8.54	23869.2	*O. sativa*	↑
NO. 18	gi|35211352	Unknown	11.42	43495.3	*G. violaceus*	↓
NO. 19	gi|30408003	NBS-type resistance protein	9.73	19422.6	*M. esculenta*	↑
NO. 20	gi|67004037	Unknown	5.48	155305.5	*O. sativa*	↓

Notes: Expression ratio was calculated relative to protein level in control sample. The “↑” indicated that the amount of protein expressed in treatment sample was greater than that in control sample; conversely, “↓” indicated that the protein expression was downregulated under *S. scitamineum* stress. The “+” indicated it was a newly induced protein after *S. scitamineum* stress.

## References

[1] Torres MA, Jones JDG, Dangl JL (2006). Reactive oxygen species signaling in response to pathogens. *Plant Physiology*.

[2] Legaz ME, de Armas R, Piñón D, Vicente C (1998). Relationships between phenolics-conjugated polyamines and sensitivity of sugarcane to smut (*Ustilago scitaminea*). *Journal of Experimental Botany*.

[3] Piñon D, de Armas R, Vicente C, Legaz ME (1999). Role of polyamines in the infection of sugarcane buds by *Ustilago scitaminea* spores. *Plant Physiology and Biochemistry*.

[4] de Armas R, Santiago R, Legaz ME, Vicente C (2007). Levels of phenolic compounds and enzyme activity can be used to screen for resistance of sugarcane to smut (*Ustilago scitaminea*). *Australasian Plant Pathology*.

[5] Orlando BH, Thomma BP, Carmona E (2005). Identification of sugarcane genes induced in disease-resistant somaclones upon inoculation with *Ustilago scitaminea* or *Bipolaris sacchari*. *Plant Physiology and Biochemistry*.

[6] Lao M, Arencibia AD, Carmona ER (2008). Differential expression analysis by cDNA-AFLP of *Saccharum* spp. after inoculation with the host pathogen *Sporisorium scitamineum*. *Plant Cell Reports*.

[7] Que YX, Yang ZX, Xu LP, Chen RK (2009). Isolation and identification of differentially expressed genes in sugarcane infected by *Ustilago scitaminea*. *Acta Agronomica Sinica*.

[8] Zhu YL, Wu JS, Wang JS (2000). Analysis of resistance-related proteins in rice against *Xanthomonas oryzae* pv. *oryzae* by two-dimensional electrophoresis. *Scientia Agricultura Sinica*.

[9] Mehta A, Rosato YB (2001). Differentially expressed proteins in the interaction of *Xanthomonas axonopodis* pv. *citri* with leaf extract of the host plant. *Proteomics*.

[10] Rep M, Dekker HL, Vossen JH (2002). Mass spectrometric identification of isoforms of PR proteins in xylem sap of fungus-infected tomato. *Plant Physiology*.

[11] Zhou WC, Kolb FL, Riechers DE (2005). Identification of proteins induced or upregulated by *Fusarium* head blight infection in the spikes of hexaploid wheat (*Triticum aestivum*). *Genome*.

[12] Zhou W, Eudes F, Laroche A (2006). Identification of differentially regulated proteins in response to a compatible interaction between the pathogen *Fusarium graminearum* and its host *Triticum aestivum*. *Proteomics*.

[13] Qian XH, He FC (2003). *Proteomics: Theory and Methods [M]*.

[14] Bradford MM (1976). A rapid and sensitive method for the quantitation of microgram quantities of protein utilizing the principle of protein dye binding. *Analytical Biochemistry*.

[15] Candiano G, Bruschi M, Musante L (2004). Blue silver: a very sensitive colloidal Coomassie G-250 staining for proteome analysis. *Electrophoresis*.

[16] Graham TL, Graham MY (1991). Cellular coordination of molecular responses in plant defense. *Molecular Plant-Microbe Interactions*.

[17] Hopkins CM, White FF, Choi SH, Guo A, Leach JE (1992). Identification of a family of avirulence genes from *Xanthomonas oryzae* pv. *oryzae*. *Molecular Plant-Microbe Interactions*.

[18] Ruan SL, Ma HS, Wang SH (2006). Advances in plant proteomics. Application of proteome techniques to plant biology research. *Hereditas*.

[19] Lee S, Lee EJ, Yang EJ (2004). Proteomic of identification of annexins, calcium-dependent membrane binding proteins that mediate osmotic stress and abscisic acid signal transduction in arabidopsis. *Plant Cell*.

[20] Eilers M, Schatz G (1986). Binding of a specific ligand inhibits import of a purified precursor protein into mitochondria. *Nature*.

[21] He BK, Li DQ (2002). Research progress in plant osmotins. *Biotechnology Bulletin*.

[22] Chang PFL, Cheah KT, Narassimhan ML, Hasegawa P, Bressan RA (1995). Osmotin gene expression is controlled by elicitor synergism. *Physiology Plant*.

[23] Lawrence GJ, Finnegan EJ, Ayliffe MA, Ellis JG (1995). The L6 gene for flax rust resistance is related to the *Arabidopsis* bacterial resistance gene *RPS2* and the tobacco viral resistance gene *N*. *Plant Cell*.

[24] Bent AF (1996). Plant disease resistance genes: function meets structure. *Plant Cell*.

[25] Traut TW (1994). The functions and consensus motifs of nine types of peptide segments that form different types of nucleotide-binding sites. *The European Journal of Biochemistry*.

[26] Chen WJ, Zhao GW, Gu YH (1999). Advance of ribulose-1, 5-bisphosphate carboxylase/oxygenase (RubisCO). *Progress in Biochemistry and Biophysics*.

[27] DeRocher EJ, Quigley F, Mache R, Bohnert HJ (1993). The six genes of the Rubisco small subunit multigene family from Mesembryanthemum crystallinum, a facultative CAM plant. *Molecular and General Genetics*.

[28] Bassi R, Rigoni F, Giacometti GM (1990). Chlorophyll binding proteins with antenna function in higher plants and green algae. *Photochemistry and Photobiology*.

[29] He XY, Zhang CH, Yang YM, Xu CN, Liu GQ (2006). Cloning and expression analysis of GhMAP1-LC3 gene from cotton (*Gossypium hirsutum* L.). *Acta Agronomica Sinica*.

[30] Mert-Türk F (2002). Phytoalexins: defence or just a response to stress?. *Journal of Cell and Molecular Biology*.

